# Accuracy of urine flow cytometry and urine test strip in predicting relevant bacteriuria in different patient populations

**DOI:** 10.1186/s12879-021-05893-3

**Published:** 2021-02-25

**Authors:** Christian Gehringer, Axel Regeniter, Katharina Rentsch, Sarah Tschudin-Sutter, Stefano Bassetti, Adrian Egli

**Affiliations:** 1 University Hospital Basel, Division of Internal Medicine, University of Basel, Basel, Switzerland; 2 University Hospital Basel, Division of Clinical Bacteriology and Mycology, University of Basel, Petersgraben 4, 4031 Basel, Switzerland; 3 University Hospital Basel, Department of Clinical Research, University of Basel, Basel, Switzerland; 4 Current affiliation: Medica Medical Laboratories Dr. F. Käppeli, Wolfbachstrasse 17, Zurich, Switzerland; 5 University Hospital Basel, Division of Clinical Chemistry, University of Basel, Basel, Switzerland; 6 Infectious Diseases and Hospital Epidemiology, University Hospital Basel, University of Basel, Basel, Switzerland; 7 Applied Microbiology Research, Department of Biomedicine, University of Basel, Basel, Switzerland

**Keywords:** Urinary tract infection, Diagnosis, Flow Cytometry, Urinalysis, Bacteria count, Urine test strip

## Abstract

**Background:**

Urinary tract infection (UTI) is diagnosed combining urinary symptoms with demonstration of urine culture growth above a given threshold. Our aim was to compare the diagnostic accuracy of Urine Flow Cytometry (UFC) with urine test strip in predicting bacterial growth and in identifying contaminated urine samples, and to derive an algorithm to identify relevant bacterial growth for clinical use.

**Methods:**

Species identification and colony-forming unit (CFU/ml) quantification from bacterial cultures were matched to corresponding cellular (leucocytes/epithelial cells) and bacteria counts per μl. Results comprise samples analysed between 2013 and 2015 for which urine culture (reference standard) and UFC and urine test strip data (index tests, Sysmex UX-2000) were available.

**Results:**

47,572 urine samples of 26,256 patients were analysed. Bacteria counts used to predict bacterial growth of ≥10^5^ CFU/ml showed an accuracy with an area under the receiver operating characteristic curve of > 93% compared to 82% using leukocyte counts. The relevant bacteriuria rule-out cut-off of 50 bacteria/μl reached a negative predictive value of 98, 91 and 89% and the rule-in cut-off of 250 bacteria/μl identified relevant bacteriuria with an overall positive predictive value of 67, 72 and 73% for microbiologically defined bacteriuria thresholds of 10^5^, 10^4^ or 10^3^ CFU/ml, respectively. Measured epithelial cell counts by UFC could not identify contaminated urine.

**Conclusions:**

Prediction of a relevant bacterial growth by bacteria counts was most accurate and was a better predictor than leucocyte counts independently of the source of the urine and the medical specialty ordering the test (medical, surgical or others).

**Supplementary Information:**

The online version contains supplementary material available at 10.1186/s12879-021-05893-3.

## Background

Urinary tract infections (UTI) are among the most common infections, both in the outpatient [1] and hospital setting [2]. They directly prolong hospital stays [3], contribute considerably to health care costs [4], increase antibiotic use, thus contributing to high levels of antibiotic resistances in uro-pathogens [5], and ultimately increase mortality [6].

The generally accepted definition of UTI includes both compatible symptoms and the presence of bacteria in urine (bacteriuria) [7]. The amount of bacteria that defines a” relevant bacterial growth” from urine culture is typically 10^5^ or more colony forming units (CFU) per ml of up to two bacterial species, although lower thresholds exist in particular for vulnerable patients or sterile sampling conditions [7, 8]. As clinical symptoms have low sensitivity and specificity, the reference method, urine culture, is necessary for a definite diagnosis of bacteriuria. However, this usually takes more than 24 h.

Rapidly available urine tests that measure surrogates of infection on test strips or count microorganisms (as in urine flow cytometry [UFC]) are utilized to anticipate the pending microbiological test result to support the diagnosis of UTI, and to guide the decision on whether to start antibiotic treatment or not.

However, their interpretation is controversial with many studies reporting different predictive values that depend on the patient collective and the defined threshold for positivity of urine cultures [9, 10].

The aim of the present study was to compare the diagnostic accuracy of UFC to urine test strip in predicting relevant bacterial growth in urine culture, and to provide easy to apply interpretation guidance based on different thresholds and patients populations.

## Methods

### Setting and study population

This retrospective study includes all specimens submitted for urine culture from January 2013 to December 2015 to the Division of Clinical Bacteriology and Mycology of the University Hospital Basel, Switzerland, a 855-bed tertiary care center with approximately 35,000 admissions per year and different outpatient departments. Outpatient departments include a general internal medicine walk-in-clinic, a family medicine clinic and specialist clinics. The study also includes all submitted samples from paediatric patients of the University Children’s Hospital Basel.

Most urine specimens submitted for culture are routinely analysed by test strip and UFC in the same ISO/IEC 17025 accredited laboratory facilities. We included all urine samples for which complete results from UFC, test strip and urine cultures were available.

### Ethics statement

Data access, processing and analysis for this study were approved by the local ethics committee (Ethikkommission Nordwest- und Zentralschweiz, Switzerland, EKNZ Nr. 2016–01534) at the request of the authors. The need of a patient informed consent was waived by the ethics committee in accordance with Swiss law because the study included only anonymised laboratory test results.

### Sample collection and preparation

Urine was collected in sterile containers that were submitted to the laboratories usually within two hours. According to local standard of practice, each urine sample was divided right after collection into two vials, one for bacterial culture (stabilized with boric acid, Urine-Monovette, Sarstedt) and the other for test strip and flow cytometry analysis (without stabilizers, Urine-Monovette, Sarstedt). Clean voided midstream urine was the preferred mode of urine collection (without prior peri-urethral cleaning). Sample collection and preparation was similar for all departments (including outpatients).

### Test strip and flow cytometry

Both UFC and test strip were automatically analysed by UX-2000 (Sysmex, Kobe, Japan) flow cytometry instruments. Test strip parameters were measured with the dual-wavelength reflectance method applied to test strips (Meditape® II 9 U, Sysmex, Kobe, Japan). The parameters included in this analysis are: nitrite, leucocyte-esterase activity (semiquantitative: positive test results above 25 leukocytes/μl) and hemoglobin/myoglobin (peroxidase-based reaction that is interpreted as positive at values above 0.03 mg/dl, precision according to manufacturer specifications).

Cellular (leukocytes/erythrocytes) and bacteria counts per μl urine were obtained from the flow cytometer unit of the UX-2000 (identical to the previous model, the UX-1000i). The UX-2000 consecutively identifies and counts leukocytes, erythrocytes (both with a linear range of 1–5000 /μl with +/− 10% precision) and epithelial cells (a sum of squamous and small round cell counts, 1–200 /μl linear range with +/− 30% precision) in the fluorescent polymethine dyed urine (the dye binds to DNA). Bacteria are quantified using the same dye but in a separate process (5–10′000 /μl linear range with +/− 20% precision). The analysis ranges of flow cytometry and test strip indicated are those supplied by the manufacturer.

UFC was performed as soon as samples were available in the laboratory and results were automatically reported in the laboratory information system.

### Bacterial cultures for quantification and species identification

Microbiological growth was assessed by inoculation of blood-agar and non-selective CHROMagar plates (bioMérieux, Lyon, France) with 1 μl urine stabilized by boric acid. Inoculation was performed in batches thrice during business hours. Both plates were usually quantified twice at 24 h and 48 h of aerobic incubation at 37 °C. Bacterial quantification was performed by experienced and trained technicians. Their judgment was based on accredited standard operating procedures to account for reproducibility. Representative colonies of dominant bacteria were identified on a matrix-assisted laser desorption/ionization time-of-flight mass spectrometer (Bruker Biotyper, Germany) or using biochemical profiling on a Vitek 2 system (bioMérieux SA, Lyon, France).

“Relevant bacteriuria/bacterial growth” was defined, in accordance with the UTI definition of the Centers for Disease Control and Prevention, as urine containing no more than two identified species of which at least one is detected in an amount of 10^5^ CFU/ml or more [7]. Lower thresholds of relevant bacteriuria (i.e. 10^4^ and 10^3^) were used to calculate test performances when indicated. Samples were defined as contaminated when microbiological cultures yielded polymicrobial growth without a dominant species.

### Data analysis and algorithm derivation

The diagnostic accuracy and predictive values of UFC and urine test strip were compared with the reference standard of bacterial culture. Combinations of tests with discrete results were linked by Boolean operators to test improvements in accuracy performances. Sensitivity, specificity, positive and negative predictive values were calculated for representative cut-offs (selected based on previously published data for comparison and around optimal cut-offs from receiver operator characteristic (ROC) curves). ROC curves and their respective areas under the curves (AUC) were calculated in R 3.3.0 [11] using the pROC package [12] to determine discriminative power and optimal, unweighted cut-offs. Analyses were stratified according to sex and hospital divisions. Calculated optimal cut-offs from ROC curves were used to derive a diagnostic algorithm to predict relevant bacteriuria. To estimate the robustness of the developed algorithm, the diagnostic accuracy of the selected cut-offs were calculated in a repeated 10-fold-split (of equal size) cross validation approach of the whole dataset using the caret package [13], with 10 independent repetitions.

## Results

Test results of 47,572 urine samples from 26,256 individual patients were analysed, representing 72% of all urine cultures performed during the study period. A total of 18,403 available microbiological results were excluded from the analysis as respective results of UFC and/or urine test strip were missing (Supplementary Fig. S1).

The samples and respective patient characteristics are summarized in Table 1. A quarter of all analyses revealed relevant bacteriuria (≥10^5^ CFU/ml) **(**Supplementary Table S1**)**. Samples of male patients had higher leukocyte but lower bacteria counts than samples of female patients (Supplementary Fig. S2 and Supplementary Table S2).

**Table 1:** Patient characteristics and sample information available for the analysed samples

Patient characteristics	n (% total)
Female	24,923 (52%)
Male	22,649 (48%)
Age range	
0–20	3593 (8%)
20–40	7513 (16%)
40–60	9716 (20%)
60–80	17,263 (36%)
80+	9487 (20%)
**Institution ordering test**	
University Hospital Basel	43,182 (91%)
- Emergency department and outpatient department	21,496 (45%)
- Medicine	7968 (17%)
- Surgery	7580 (16%)
- Intensive care	2984 (6%)
- Gynaecology/Obstetrics	3154 (7%)
Children’s Hospital Basel	3305 (7%)
Physicians, resident homes and others	1048 (2%)
Unknown	37 (0%)
**Material Category**	
Midstream	31,157 (65%)
One-time catheter	4456 (9%)
Indwelling catheter	7696 (16%)
Other sources^1^	4263 (9%)
**Total**	**47,572 (100%)**

1) Includes urine from suprapubic catheters, not further specified samples, unknown, before/after manual stimulation of prostate, urine bags and other

*Escherichia coli* was the most frequently identified microorganism, followed by *Klebsiella spp.*, *Lactobacillus spp.* and *Enterococcus faecalis* (Supplementary Table S3).

Predictive values of UFC and test strips were calculated for all samples and different microbiological thresholds of relevant bacteriuria (Table 2), and individually for both sexes and across all different medical specialties and ordering institutions respectively (Supplementary Table S4). Bacteria counts measured by urine flow cytometry showed the best discriminatory power to identify relevant bacteriuria. Only the detection of nitrate proved to be unmatched in its specificity (98%), however, revealed a poor sensitivity of below 40%. Leukocyte counts using a cut-off of about 30–40 leukocytes reached predictive values comparable to leukocyte esterase detection on test strips. Comparison of relevant microbial growth between different species revealed an overall higher sensitivity of bacteria counts in detecting gram-positive species where test strips performed poorly (Supplementary Table S5).

**Table 2:** Sensitivity (SENS), specificity (SPEC), positive and negative predictive values (PPV and NPV), and numbers of false negative (FN) and false positive results (FP) when test strips or flow cytometry was used to separate relevant bacteriuria from bacteriuria, contamination or no culture growth of all samples using different cut-off values. Test performances for different definitions of relevant bacteriuria (≥ 10^5^, ≥ 10^4^ and ≥ 10^3^, respectively) are listed. Pos.: positive test strip result

Bacterial growth (CFU/ml)	Method	Test Cut-off		SENS (%)	SPEC (%)	PPV (%)	NPV (%)	FN	FP
≥ 10^5^	Test strip	Nitrate	pos.	35.8	97.9	84.9	82.0	7662	758
		Leuk. esterase	pos.	81.5	68.1	46.1	91.7	2211	11,372
	Flow cytometry	Leukocyte count	10	91.8	46.2	36.3	94.4	981	19,190
			50	75.9	74.7	50.1	90.2	2876	9028
		Bacteria count	50	95.8	67.8	49.9	98.0	497	11,471
			250	87.2	85.6	66.9	95.2	1528	5137
≥ 10^4^	Test strip	Nitrate	pos.	28.4	98.1	87.8	73.8	11,129	613
		Leuk. esterase	pos.	75.0	70.5	55.2	85.3	3892	9441
	Flow cytometry	Leukocyte count	10	87.2	48.2	45.0	88.6	1986	16,583
			50	68.8	76.9	59.2	83.6	4844	7384
		Bacteria count	50	86.3	70.4	58.6	91.4	2122	9484
			250	72.4	86.6	72.4	86.6	4295	4292
≥ 10^3^	Test strip	Nitrate	pos.	27.2	98.1	88.2	72.2	11,836	592
		Leuk. esterase	pos.	74.1	71.1	57.2	84.1	4213	9034
	Flow cytometry	Leukocyte count	10	86.8	48.8	46.8	87.7	2149	16,018
			50	68.1	77.6	61.2	82.4	5197	7009
		Bacteria count	50	83.8	70.4	59.6	89.3	2628	9262
			250	69.6	86.5	72.9	84.6	4949	4218

**Fig. 1:**
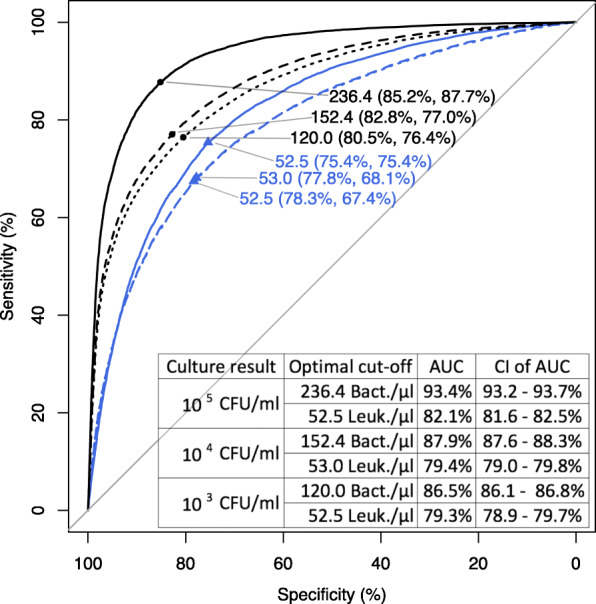
Receiver operating characteristic (ROC) curves of bacteria (black) and leukocyte counts (blue, triangles) with the respective optimal calculated, unweighted cut-offs (and the respective specificity/sensitivity in brackets) for 10^3^ CFU/ml (dotted lines), 10^4^ CFU/ml (dashed lines) and 10^5^ CFU/ml (solid lines) and corresponding areas under the ROC curves (AUC) indicated in the table including confidence intervals (CI). Note that 10^3^ CFU/ml and 10^4^ CFU/ml ROC curves of leukocyte counts overlap with almost identical progression

Measures of discrimination were visualized by ROC curves for bacteria count and leukocyte count as predictors of positive urine culture (Fig. 1). Lowering the threshold for the definition of a relevant bacteriuria to 10^4^ or 10^3^ CFU/ml resulted in lower optimal unweighted cut-offs in a non-linear progression for bacteria counts, while leukocyte counts did not show similar dynamics. The bacteria count ROC curves progressions were very similar (AUC between 93 and 95%) for all but the gynaecology & obstetrics division (AUC: 86.4%, Supplementary Fig. S3); ROC curves of leukocyte count test progressions resulted in wider dynamics (AUC between 74.0 and 83.6%).

Whole epithelial cell count (as determined by UFC) appeared to be a poor predictor of contaminated samples with a optimal cut-off value reaching a sensitivity of around 60% and a specificity of 50%, regardless of whether contaminations included all polymicrobial samples or additionally all samples with bacterial growth below 10^5^ CFU/ml (Supplementary Fig. S4 and Supplementary Table S6).

**Fig. 2:**
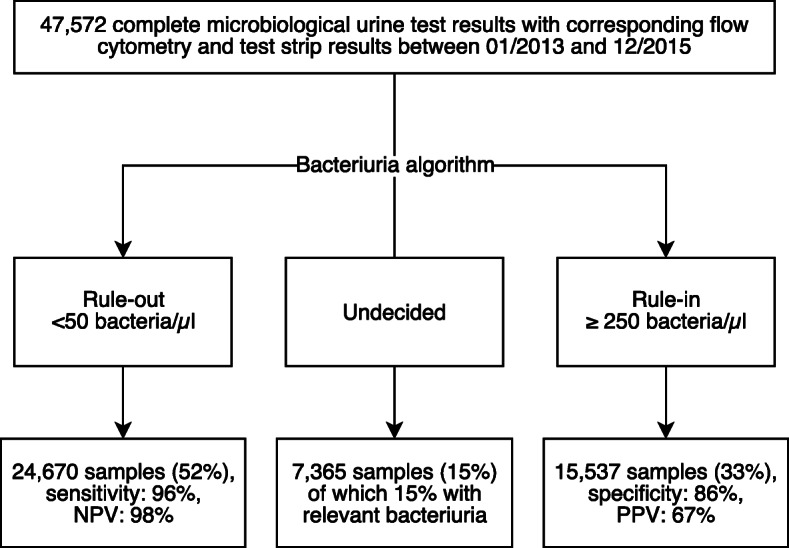
Theoretical performance of the developed bacteriuria algorithm on the whole data set. Relevant bacteriuria: samples of 1–2 identified bacteria of ≥10^5^ colony-forming unit/ml, NPV: negative predictive value, PPV: positive predictive value

We developed an algorithm based on bacteria count (Fig. 2). To rule out relevant bacteriuria, a cut-off of 50 bacteria/μl was selected based on the sensitivity and the negative predictive value of > 95%. To rule in relevant bacteriuria, a cut-off of 250 bacteria/μl was chosen to improve specificity across a variety of patient characteristics. The performance of the algorithm when applied to the whole data set and all subgroups is summarized in Table 3. The selected cut-offs demonstrated minor variations (mostly around or below 1% change from the median) of the predictive values in a cross validation approach (Supplementary Fig. S5 and Supplementary Table S7).

**Table 3:** Performance of the developed algorithm to detect relevant bacteriuria (≥ 10^5^ colony-forming units/ml) for all samples, samples of male and female patients and for samples of different divisions, respectively

	All samples (47,572)	Female (24,923)	Male (22,649)	Emergency& Outpatient (21,496)	Medicine (7968)	Surgery (7580)	Intensive care (2984)	Gynaecology & Obs. (3154)	Paediatrics (3305)	External (1048)
Rule-out (< 50 bacteria/μl)										
Sensitivity, %	96	97	93	96	94	95	96	97	96	96
Negative predictive value, %	98	97	99	98	98	98	99	97	98	97
Rule-in (≥ 250 bacteria/μl)										
Specificity, %	86	76	94	83	89	91	90	69	87	85
Positive predictive value, %	67	65	73	68	69	72	59	49	69	75
Number of undecided samples, i.e. ≥ 50 and < 250 bacteria/μl (% of total within that group)	7365 (15)	4694 (19)	2671 (12)	3245 (15)	1168 (15)	1040 (13)	471 (16)	673 (21)	600 (18)	155 (15)
Relevant bacteriuria samples within undecided samples, %	14	14	14	15	15	15	9	12	13	14

## Discussion

UTI is a clinical diagnosis confirmed by the detection of relevant bacterial growth in urine culture. In this context, the finding that the growth of bacteria (by microbiological culture) can be predicted by counting them seems trivially intuitive and has been confirmed by many studies for more than one decade [10, 14]. Our data reproduce these findings and extend them to a more generalisable conclusion due to the large sample size deriving from a diverse patient population, as well as head-to-head comparisons with other same-day tests.

In our experience, for diagnosis of UTI, clinicians seem to rely more on clinical symptoms considered to be “typical”, but which are poorly predictive of UTI (such as costo-vertebral angle tenderness) [15] and on dipstick test results with poor positive predictive value (e.g. leucocytes esterase) [9], rather than on results of UFC. This is possibly due to uncertainty associated with the interpretation of UFC results. We therefore focused on the clinical applicability of our results to predict bacteriuria as opposed to most studies on UFC, which are centered on the laboratory efficacy and efficiency by reducing bacterial culture and pre-selection of samples. To the best of our knowledge, this study represents the largest published dataset applied to compare the diagnostic accuracy of UFC with urine test strip for prediction of bacterial growth in urine culture in both hospital and community settings.

Calculated predictive values were very similar for bacteria counts in all subgroups except the gynaecology & obstetrics division. The dominance of samples from female patients in this division can partly explain the deviation since separate analysis of samples from all female patients across subgroups (Supplementary Fig. S6) showed a similar ROC curve progression. Additionally, a different pre-test probability is likely to influence the performance of samples from this particular division. In our study, samples from male patients had higher leukocyte counts but lower bacteria counts than from female patients. This observation was previously made and might be explained by physiological differences [16, 17].

Our analysis includes unselected samples but, due to the study design, lacks any patient-specific clinical information or information on antibiotic therapies; study results consequently only apply to the diagnosis of bacteriuria and not the clinical diagnosis of UTI. The diagnostic performance, however, is similar to a recent smaller study that included clinical symptoms and focused on sensitivity at a 10^4^ bacteria/μl threshold (Fig. 1) [18]. An installed antibiotic therapy before sample taking might have decreased overall specificity of both, bacteria counts (counting of non-viable bacteria) as well as leucocyte counts (counting of leucocytes that persisted in the urinary tract even after bacteria have been eradicated, i.e. no bacterial growth on culture media). Since urine analysis is usually not recommended after the initiation of therapy and unidirectionally influences every test, we expect this lack of information not to limit the generalisable of our test comparison.

Approximately 28% of available microbiologically analysed urine samples were excluded since they lacked a corresponding UFC and/or test strip result. They consisted partly of urine from external sources or did not have the quantity required to perform both tests. We cannot exclude a possible bias based on this pre-selection.

We did not find a combination of two different diagnostic tests that improved the predictive values significantly (exemplified with leukocyte esterase and nitrate in Supplementary Table S4). This is not unexpected given the fact that those tests are conditionally dependent on the presence of bacteria and infection [19]. The combination of leukocyte esterase and nitrate only improved sensitivity if test performance was analysed on a species level (Supplementary Table S5). Other more complex combinations of more than two tests, in particular when the continuity of UFC variables is utilized in contrast to fixed cut-off values, might lead to improved optima of predictive values but would be more challenging to clinically interpret if not automatically integrated in the laboratory reporting system.

We did not use the bacterial morphology software that tries to distinguish gram-positive from mixed (gram-positive/gram-negative) bacteria since our initial analysis seemed to confirm insufficient performance as described earlier [20]. Similarly, erythrocytes measured by flow cytometry were neglected in our analysis since they might support primarily other urological disease diagnoses and seemed only of inferior importance for UTI, although we recognize that hematuria might be used as a sign for UTI as well.

In the context of urinary tract infection, squamous epithelial cells determined by microscopy are traditionally used to identify contaminated samples. The evidence supporting this correlation is very scarce [21] but even if valid, “epithelial cell count” from flow cytometry seems not to be a helpful parameter. According to the user manual (Sysmex flow cytometer UX-2000), depending on the setup of the instrument, the “epithelial cell count” does not include only squamous epithelial cells but is a group parameter for squamous and non-squamous epithelial cells and small round cells [22]. Small round cells “[...] *include renal tubular epithelial cells, squamous epithelial cells, middle and deep layer of transitional epithelial cells [...]”* [22]*.* In our study, the epithelial cell count by UFC was not useful to predict urine contamination and performed only slightly better than chance in this binary decision.

The calculated cut-offs seem suitable for our setting, are independent of the clinical department and robust after internal validation. Despite similar cut-offs from other studies (using identical flow cytometers) external validation is needed in order to generalise our results as are modifications of the cut-offs when instrument development progresses.

The diagnostic algorithm developed as a prediction tool for relevant bacterial growth contains two distinctive cut-offs, one optimized for sensitivity and the other for specificity, which resulted in less dependency on patient characteristics. Under the assumption of a stable, uncritical patient, a urine sample of the “undecided” group might direct the clinician to wait for bacterial culture confirmation before initiating an empiric therapy. On the other hand, the rule-in cut-off might be enough to initiate a therapy if symptoms are present.

This algorithm might help clinicians to estimate the microbiological culture result with high sensitivity and robustness and acceptable specificity and therefore improve adequate antibiotic use. Ultimately, the prognostic performance of the presented algorithm can only be evaluated by a prospective study that includes clinical parameters and allows to distinguish UTI rather than bacteriuria and to estimate the influence of the diagnostic test on therapies and other health-related outcomes.

## : Conclusions

The present study showed that bacteria counts measured by UFC offer a better diagnostic accuracy for the prediction of bacterial growth in urine cultures than leukocyte count or nitrate and leukocyte esterease on test strips. Our results were used to derive a simple algorithm for clinical practice based on bacteria count alone. Epithelial cells measured by UFC did not contribute to identifying contaminated urine samples.

## Supplementary Information

**Additional file 1.**

## Data Availability

Data sharing was not approved by the local ethics committee as the large datasets analysed during the current study could potentially be used for individual identification albeit being anonymised.

## Abbreviations

UTI: Urinary tract infection
CFU: Colony-forming unit
UFC: Urine Flow Cytometry
ROC: Receiver operator characteristic (curve)
AUC: Area under the (ROC) curve
