# Supplementary material for: Accuracy of urine flow cytometry and urine test strip in predicting relevant bacteriuria in different patient populations
Source: BMC Infect Dis. 2021 Feb 25;21:209. doi: 10.1186/s12879-021-05893-3 (PMC7908726; doi:10.1186/s12879-021-05893-3)
Supplement: Supplementary file 1 — Additional file 1. [file 12879_2021_5893_MOESM1_ESM.docx]

Supplementary Figures and Tables for

**Accuracy of urine flow cytometry and urine test strip in predicting relevant bacteriuria in different patient populations**

Christian Gehringer^1,2,3^, Axel Regeniter^4^, Katharina Rentsch^5^, Sarah Tschudin-Sutter^6^, Stefano Bassetti^1,3*^, Adrian Egli^2,3,7*^

1) University Hospital Basel, Division of Internal Medicine, University of Basel, Basel, Switzerland

2) University Hospital Basel, Division of Clinical Bacteriology and Mycology, University of Basel, Basel, Switzerland

3) University Hospital Basel, Department of Clinical Research, University of Basel, Basel, Switzerland

4) Current affiliation: Medica Medical Laboratories Dr. F. Käppeli, Wolfbachstrasse 17, CH-8032 Zurich, Switzerland

5) University Hospital Basel, Division of Clinical Chemistry, University of Basel, Basel, Switzerland

6) Infectious Diseases and Hospital Epidemiology, University Hospital Basel, University of Basel, Basel, Switzerland

7) Applied Microbiology Research, Department of Biomedicine, University of Basel, Basel, Switzerland

Keywords: Urinary tract infection, Bacteriuria, Urine flow cytometry

* contributed equally to this work

Corresponding author

Prof. Adrian Egli, FAMH

Head of Division, Clinical Bacteriology and Mycology

University Hospital Basel

Petersgraben 4

4031 Basel

Phone: +41 61 556 57 49

Secretary: +41 61 265 42 44

adrian.egli@usb.ch

Supplementary Figure S 1 Sample set formation and composition of data set based on microbiological test results. Relevant bacteriuria: samples of 1-2 identified bacteria of ≥10^5^ colony-forming units/ml. 5

Supplementary Figure S 2 Flow cytometry count ranges separated by diagnostic groups categorization based on bacterial growth. Numbers in brackets indicate total number of samples within each group. All samples (A, D) were split into samples from male (B, E) and female patients (C, F). 7

Supplementary Figure S 3 Receiver operating characteristic (ROC) curves of bacteria (continuous lines) and leukocyte counts (dotted lines) with the respective optimal calculated, unweighted cut-offs (and the respective specificity/sensitivity in brackets) and area under the ROC curves for all samples and for sub-analysis by the respective divisions ordering the test. 18

Supplementary Figure S 4 Receiver operating characteristic (ROC) curves of epithelial cell counts measured by flow cytometry with the respective optimal calculated, unweighted cut-offs (and the respective specificity/sensitivity in brackets) when used to discriminate all contaminated samples (polymicrobial growth without a dominant species, blue solid line, triangle) and all contaminated samples and additionally all samples with growth under 10^5^ colony forming units (black solid line, circle). 20

Supplementary Figure S 5 Performance of accuracy and predictive variables of the cut-offs selected in the relevant bacteriuria algorithm when assessed in the 10 times repeated 10-fold cross validation approach. 21

Supplementary Figure S 6 Receiver operating characteristic (ROC) curves of bacteria and leukocyte counts with the respective optimal calculated, unweighted cut-offs (and the respective specificity/sensitivity in brackets) for all samples, samples of male patients and samples of female patients. 23

Supplementary Table S 1 Categorization of all analysed samples based on the reference standard of bacterial growth differentiated by sample type and patient characteristics. 6

Supplementary Table S 2 Medians and ranges (1^st^ and 3^rd^ quartiles) of bacteria and leukocyte counts measured by urine flow cytometry from all samples of relevant bacteriuria (≥ 10^5^colony-forming units/ml) and separated for samples of female and male patients. 8

Supplementary Table S 3 Species found in samples grouped by maximum bacterial concentration (≥ 10^5^CFU/ml or < 10^5^ CFU/ml) and total number of identified organisms, or mixed flora, respectively. 9

Supplementary Table S 4 Sensitivity (SENS), specificity (SPEC), positive and negative predictive values (PPV and NPV), and numbers of false negative (FN) and false positive results (FP) when test strips or flow cytometry was used to separate relevant bacteriuria (≥ 10^5^ CFU/ml of identified species) from bacteriuria < 10^5^ CFU/ml, contamination or no culture growth of samples separated by samples of each sex and ordering department using different cut-off values. Pos: positive test strip result. This is an extended version of Table 2. 10

Supplementary Table S 5 Individual calculation of sensitivity (SENS) and numbers of false negatives (FN) for samples of relevant monomicrobial growth (≥ 10^5^ CFU/ml of identified species) when test strips or flow cytometry was used to identify relevant bacteriuria using different cut-off values. Since species identification is not reliably available for samples of bacteriuria < 10^5^ CFU/ml (e.g. when classified as Enterobacteriaceae), specificity and other predictive values than SENS and FN are not available. Pos: positive test strip 14

Supplementary Table S 6 Predictive values of epithelial cell counts measured by urine flow cytometry (UFC, a sum of squamous and small round cell counts) when used to identify contaminated samples. Sensitivity (SENS), specificity (SPEC), positive and negative predictive values (PPV and NPV), false negative (FN) and false positive results (FP), CFU: colony-forming units. 19

Supplementary Table S 7 Sensitivity (SENS), specificity (SPEC), positive and negative predictive values (PPV and NPV) of the cut-offs selected in the relevant bacteriuria algorithm when assessed in the 10 times repeated 10-fold cross validation approach. 22

**
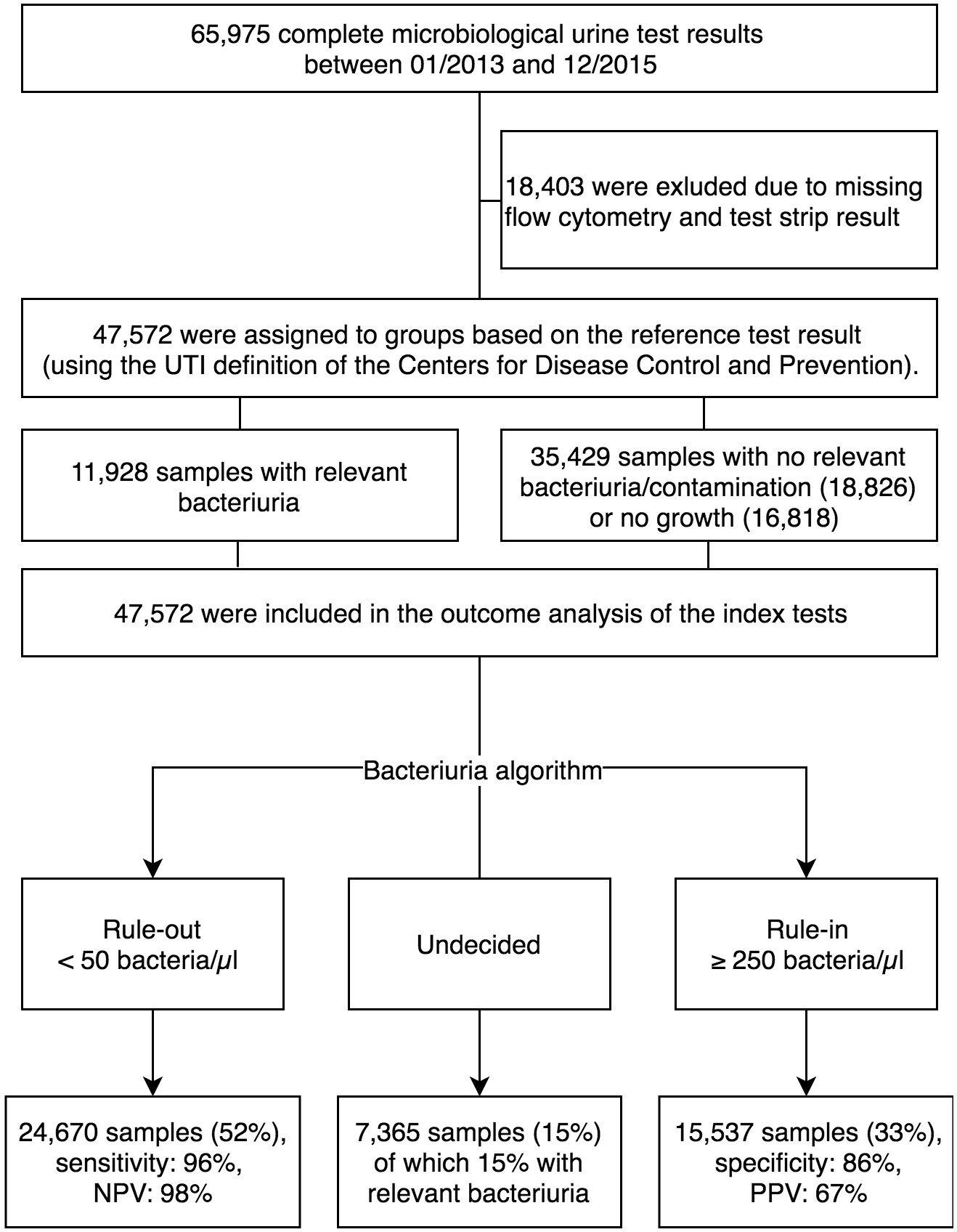
**

Supplementary Figure S 1 Sample set formation and composition of data set based on microbiological test results. Relevant bacteriuria: samples of 1-2 identified bacteria of ≥10^5^ colony-forming units/ml.

Supplementary Table S 1 Categorization of all analysed samples based on the reference standard of bacterial growth differentiated by sample type and patient characteristics.

| Microbiological result | No growth | 10^3^-10^4^ CFU/ml | | ≥ 10^5^ CFU/ml | |  |
| --- | --- | --- | --- | --- | --- | --- |
| Number of identified organisms | 0 | 0 | 1-2 | 0 | 1-2 |  |
| n (% of total) | 16818 (35%) | 10195 (21%) | 4125 (9%) | 4506 (9%) | 11928 (25%) | **47572 (100%)** |
| **Material Category** | | | | | | **n (% total)** |
| Midstream | 9,214 | 8,521 | 2,782 | 3,372 | 7,268 | 31157 (65%) |
| One-time catheter | 2,241 | 208 | 438 | 90 | 1,479 | 4456 (9%) |
| Indwelling catheter | 3,984 | 494 | 405 | 674 | 2,139 | 7696 (16%) |
| Other sources^3^ | 1,379 | 972 | 500 | 370 | 1,042 | 4263 (9%) |
| **Institution ordering test** | | | | | | |
| University Hospital Basel | |  |  |  |  | 43182 (91%) |
| - Emergency and outpatient department | 6,514 | 4,817 | 1,768 | 2,232 | 6,165 | 21496 (45%) |
| - Medicine | 2,946 | 1,790 | 703 | 741 | 1,788 | 7968 (17%) |
| - Surgery | 3,337 | 1,503 | 638 | 523 | 1,579 | 7580 (16%) |
| - Intensive care | 1,943 | 281 | 171 | 168 | 421 | 2984 (6%) |
| - Gynecology/Obstetrics | 754 | 808 | 293 | 507 | 792 | 3154 (7%) |
| Children's Hospital Basel | 1,075 | 753 | 464 | 193 | 820 | 3305 (7%) |
| Physicians, resident homes and others | 244 | 233 | 80 | 136 | 355 | 1048 (2%) |
| Unknown | 5 | 10 | 8 | 6 | 8 | 37 (0%) |
| **Patient characteristics** | | | | | | |
| Female | 5,209 | 5,514 | 2,339 | 3,497 | 8,364 | 24923 (52%) |
| Male | 11,609 | 4,681 | 1,786 | 1,009 | 3,564 | 22649 (48%) |
| Age range |  |  |  |  |  |  |
| 0-20 | 1,125 | 806 | 483 | 253 | 926 | 3593 (8%) |
| 20-40 | 2,261 | 1,801 | 688 | 991 | 1,772 | 7513 (16%) |
| 40-60 | 3,934 | 2,201 | 790 | 878 | 1,913 | 9716 (20%) |
| 60-80 | 6,662 | 3,646 | 1,474 | 1,458 | 4,023 | 17263 (36%) |
| 80+ | 2,836 | 1,741 | 690 | 926 | 3,294 | 9487 (20%) |
| 1) Effective antibiotic therapy that was started prior to sample taking may also result in no bacterial growth. 2) Bacteriuria, that meets requirements for UTI definitions (together with symptoms, which were not available in this study).  3) Includes urine from suprapubic catheters, not further specified samples, unknown, before/after manual stimulation of prostate, urine bags and other. | | | | | | |


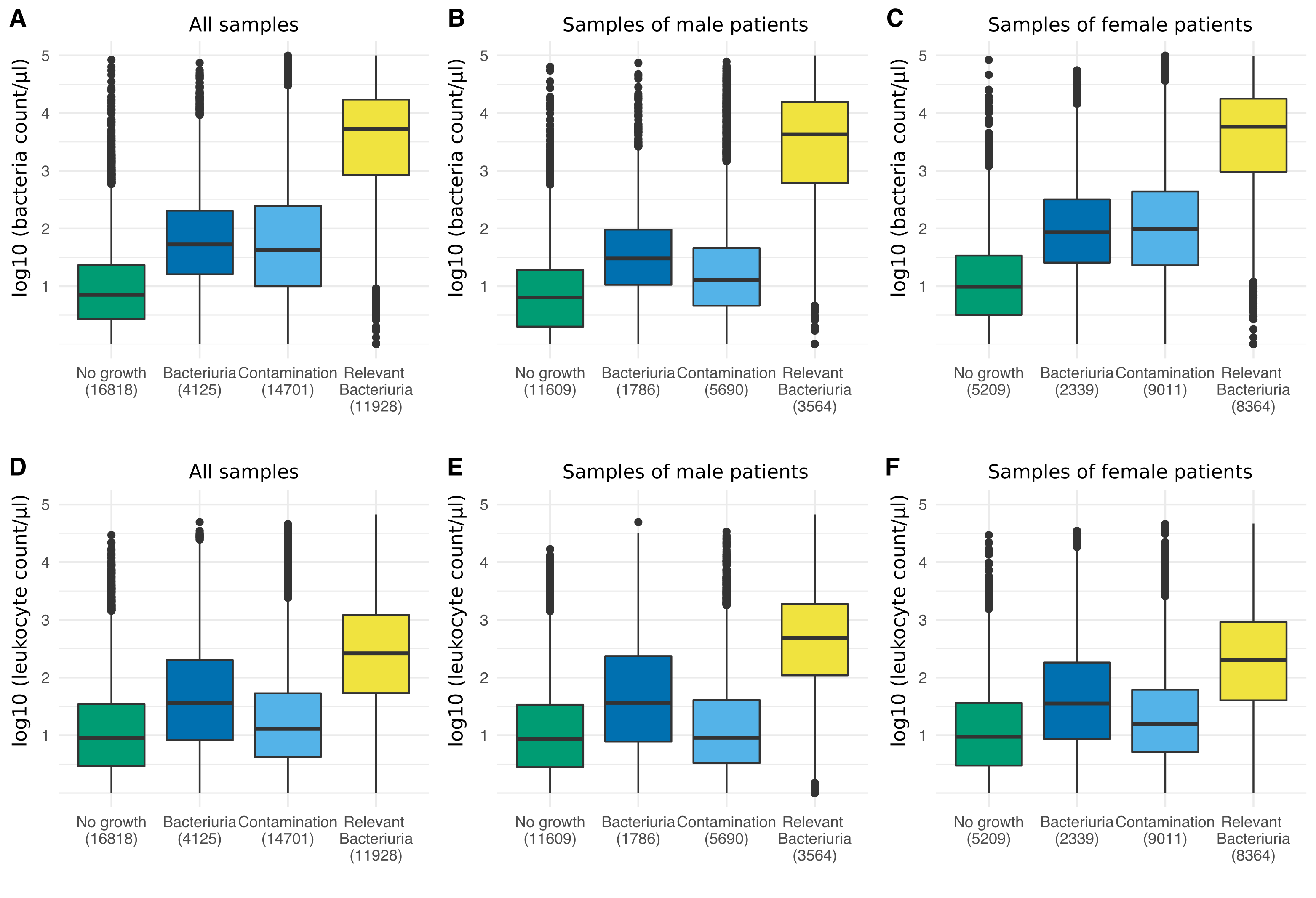


Supplementary Figure S 2 Flow cytometry count ranges separated by diagnostic groups categorization based on bacterial growth. Numbers in brackets indicate total number of samples within each group. All samples (A, D) were split into samples from male (B, E) and female patients (C, F).

Supplementary Table S 2 Medians and ranges (1^st^ and 3^rd^ quartiles) of bacteria and leukocyte counts measured by urine flow cytometry from all samples of relevant bacteriuria (≥ 10^5^colony-forming units/ml) and separated for samples of female and male patients.

| Method | All samples | Female only | Male only |
| --- | --- | --- | --- |
| Leukocyte count/µl | 257 (49 – 1,250) | 200 (37 - 965) | 483 (96 – 1,960) |
| Bacteria count/µl | 4,722 (733 – 16,709) | 5,392 (887 – 17,688) | 3,471 (485 – 14,691) |

Supplementary Table S 3 Species found in samples grouped by maximum bacterial concentration (≥ 10^5^CFU/ml or < 10^5^ CFU/ml) and total number of identified organisms, or mixed flora, respectively.

| **n** | **%** | **Dominant species, genus or group** |
| --- | --- | --- |
| **Monomicrobial samples of ≥ 10^5^ CFU/ml (n = 7,826; 7,611**, when *Candida spp*. samples are excluded**)** | | |
| 3489 | 45% | *Escherichia coli* (ESBL: n = 236, 6.8%) |
| 763 | 10% | *Klebsiella spp.* (mostly *K. pneumoniae*, ESBL: n = 42, 5.5%) |
| 715 | 9% | *Lactobacillus spp.* |
| 483 | 6% | *Enterococcus faecalis* |
| 316 | 4% | *Gardnerella spp.* |
| 223 | 3% | *Pseudomonas aeruginosa* |
| 213 | 3% | *Enterococcus faecium* |
| (215 | 3% | *Candida spp.*, categorized as contaminated urine sample for analysis) |
| **Polymicrobial samples of ≥ 10^5^ CFU/ml (n = 4,317)** | | |
| 1264 | 29% | Combinations of *Escherichia coli, Klebsiella spp., Enterococcus spp., Proteus spp., Gardnerella,* non-dominant flora, and other identified species |
| 1227 | 28% | *Escherichia coli* (ESBL: n = 33 (2.7%)) with other non-dominant flora (not further specified) |
| 990 | 23% | Combinations of *Streptococcus spp, Staphylococcus spp., Pseudomonas aeruginosa, Citrobacter spp*. and other |
| 326 | 8% | Enterobacteriacea with other non-dominant flora (not further specified) |
| 59 | 1% | *Lactobacillus spp*. with other non-dominant flora (not further specified) |
| **Mixed flora ≥ 10^5^ CFU/ml without a dominant, identified species (n = 4,291)** | | |
| **Monomicrobial samples of < 10^5^ CFU/ml (n = 3,111)** | | |
| 553 | 18% | *Escherichia coli* (ESBL: n = 46, 8.3%) |
| 378 | 12% | *Lactobacillus spp.* |
| 259 | 8% | not further specified gram positive cocci (Coagulase negative Staphylococci not *S. saprophyticus*) |
| 236 | 8% | *Enterococcus faecalis* |
| 247 | 8% | *Candida spp.* |
| 163 | 5% | *Gardnerella spp.* |
| 132 | 4% | Enterobacteriaceae (not further specified) |
| 125 | 4% | *Klebsiella spp.* (mostly *K. pneumoniae*, ESBL: n = 6, 4.8%) |
| 91 | 3% | *Enterococcus faecium* |
| 82 | 3% | *Pseudomonas aeruginosa* |
| **Polymicrobial samples of < 10^5^ CFU/ml (n = 1,014)** | | |
| 237 | 23% | *Escherichia coli* in combinations with other species |
| 165 | 16% | *Candida spp.* in combinations with other species |
| 112 | 11% | Enterobacteriaceae in combinations with other species |
| **Mixed flora < 10^5^ CFU/ml without a dominant, identified species (n = 10,195)** | | |
| ESBL: Number of extended spectrum beta-lactamase resistant strains, percentage within that row  CFU: colony-forming units/ml | | |

Supplementary Table S 4 Sensitivity (SENS), specificity (SPEC), positive and negative predictive values (PPV and NPV), and numbers of false negative (FN) and false positive results (FP) when test strips or flow cytometry was used to separate relevant bacteriuria (≥ 10^5^ CFU/ml of identified species) from bacteriuria < 10^5^ CFU/ml, contamination or no culture growth of samples separated by samples of each sex and ordering department using different cut-off values. Pos: positive test strip result. This is an extended version of Table 2.

|  | **Method** | **Test Cut-off** | | **SENS (%)** | **SPEC (%)** | **PPV (%)** | **NPV (%)** | **FN** | **FP** |
| --- | --- | --- | --- | --- | --- | --- | --- | --- | --- |
| All samples (n=47572) | Test strip | Nitrate | pos. | 35.8 | 97.9 | 84.9 | 82.0 | 7,662 | 758 |
|  |  | Leuk. esterase | pos. | 81.5 | 68.1 | 46.1 | 91.7 | 2,211 | 11,372 |
|  |  | Nitrate or Leukocyte esterase | pos. | 84.3 | 67.8 | 46.7 | 92.8 | 1,870 | 11,470 |
|  | Flow cytometry | Leukocyte count | 10 | 91.8 | 46.2 | 36.3 | 94.4 | 981 | 19,190 |
|  |  |  | 40 | 78.3 | 71.8 | 48.2 | 90.8 | 2,584 | 10,059 |
|  |  |  | 50 | 75.9 | 74.7 | 50.1 | 90.2 | 2,876 | 9,028 |
|  |  |  | 100 | 65.3 | 82.8 | 55.9 | 87.7 | 4,138 | 6,139 |
|  |  |  | 200 | 55.1 | 88.3 | 61.3 | 85.5 | 5,360 | 4,153 |
|  |  | Bacteria count | 20 | 98.1 | 52.6 | 40.9 | 98.8 | 231 | 16,912 |
|  |  |  | 50 | 95.8 | 67.8 | 49.9 | 98.0 | 497 | 11,471 |
|  |  |  | 100 | 93.1 | 76.7 | 57.2 | 97.1 | 828 | 8,291 |
|  |  |  | 200 | 88.8 | 83.7 | 64.6 | 95.7 | 1,332 | 5,802 |
|  |  |  | 250 | 87.2 | 85.6 | 66.9 | 95.2 | 1,528 | 5,137 |
| Samples of female patients (n=24,923) | Test strip | Nitrate | pos. | 35.2 | 97.6 | 87.9 | 74.9 | 5,421 | 405 |
|  |  | Leuk. esterase | pos. | 78.9 | 62.0 | 51.2 | 85.3 | 1,767 | 6,296 |
|  |  | Nitrate or Leukocyte esterase | pos. | 82.5 | 61.6 | 52.0 | 87.5 | 1,464 | 6,357 |
|  | Flow cytometry | Leukocyte count | 10 | 90.4 | 41.5 | 43.8 | 89.6 | 799 | 9,695 |
|  |  |  | 40 | 75.1 | 69.0 | 55.1 | 84.6 | 2,086 | 5,126 |
|  |  |  | 50 | 72.3 | 72.3 | 56.8 | 83.8 | 2,320 | 4,590 |
|  |  |  | 100 | 60.9 | 81.1 | 62.0 | 80.4 | 3,269 | 3,128 |
|  |  |  | 200 | 50.2 | 87.3 | 66.6 | 77.6 | 4,164 | 2,108 |
|  |  | Bacteria count | 20 | 98.6 | 35.6 | 43.6 | 98.1 | 113 | 10,659 |
|  |  |  | 50 | 96.9 | 51.3 | 50.1 | 97.0 | 263 | 8,059 |
|  |  |  | 100 | 94.5 | 62.5 | 56.0 | 95.7 | 463 | 6,204 |
|  |  |  | 200 | 90.5 | 72.8 | 62.7 | 93.8 | 797 | 4,504 |
|  |  |  | 250 | 88.9 | 75.7 | 64.9 | 93.1 | 926 | 4,028 |

| Samples of male patients (n=22,649) | Test strip | Nitrate | pos. | 37.1 | 98.2 | 78.9 | 89.3 | 2,241 | 353 |
| --- | --- | --- | --- | --- | --- | --- | --- | --- | --- |
|  |  | Leuk. esterase | pos. | 87.5 | 73.4 | 38.1 | 96.9 | 444 | 5,076 |
|  |  | Nitrate or Leukocyte esterase | pos. | 88.6 | 73.2 | 38.2 | 97.2 | 406 | 5,113 |
|  | Flow cytometry | Leukocyte count | 10 | 94.9 | 50.2 | 26.3 | 98.1 | 182 | 9,495 |
|  |  |  | 40 | 86.0 | 74.2 | 38.3 | 96.6 | 498 | 4,933 |
|  |  |  | 50 | 84.4 | 76.7 | 40.4 | 96.3 | 556 | 4,438 |
|  |  |  | 100 | 75.6 | 84.2 | 47.2 | 94.9 | 869 | 3,011 |
|  |  |  | 200 | 66.4 | 89.3 | 53.7 | 93.4 | 1,196 | 2,045 |
|  |  | Bacteria count | 20 | 96.7 | 67.2 | 35.5 | 99.1 | 118 | 6,253 |
|  |  |  | 50 | 93.4 | 82.1 | 49.4 | 98.5 | 234 | 3,412 |
|  |  |  | 100 | 89.8 | 89.1 | 60.5 | 97.9 | 365 | 2,087 |
|  |  |  | 200 | 85.0 | 93.2 | 70.0 | 97.1 | 535 | 1,298 |
|  |  |  | 250 | 83.1 | 94.2 | 72.8 | 96.8 | 602 | 1,109 |
| Emergency department and outpatient department | Test strip | Nitrate | pos. | 36.1 | 97.5 | 85.1 | 79.1 | 3,941 | 388 |
|  |  | Leuk. esterase | pos. | 83.0 | 67.9 | 51.0 | 90.9 | 1,048 | 4,919 |
|  |  | Nitrate or Leukocyte esterase | pos. | 86.1 | 67.6 | 51.6 | 92.3 | 860 | 4,967 |
|  | Flow cytometry | Leukocyte count | 10 | 92.2 | 48.4 | 41.8 | 93.9 | 479 | 7,911 |
|  |  |  | 40 | 79.6 | 71.8 | 53.1 | 89.7 | 1,257 | 4,327 |
|  |  |  | 50 | 77.2 | 74.4 | 54.9 | 89.0 | 1,404 | 3,918 |
|  |  |  | 100 | 66.8 | 82.1 | 60.0 | 86.0 | 2,046 | 2,751 |
|  |  |  | 200 | 56.8 | 87.3 | 64.3 | 83.4 | 2,661 | 1,946 |
|  |  | Bacteria count | 20 | 98.5 | 51.0 | 44.7 | 98.8 | 95 | 7,507 |
|  |  |  | 50 | 96.3 | 65.3 | 52.7 | 97.8 | 228 | 5,318 |
|  |  |  | 100 | 94.0 | 74.2 | 59.4 | 96.8 | 372 | 3,952 |
|  |  |  | 200 | 89.9 | 81.4 | 66.0 | 95.2 | 623 | 2,851 |
|  |  |  | 250 | 88.4 | 83.3 | 68.0 | 94.7 | 715 | 2,560 |
| Medical | Test strip | Nitrate | pos. | 37.2 | 98.0 | 84.3 | 84.4 | 1,122 | 124 |
|  |  | Leuk. esterase | pos. | 80.4 | 72.6 | 45.9 | 92.8 | 350 | 1,695 |
|  |  | Nitrate or Leukocyte esterase | pos. | 83.2 | 72.3 | 46.5 | 93.7 | 300 | 1,712 |
|  | Flow cytometry | Leukocyte count | 10 | 91.7 | 47.3 | 33.5 | 95.2 | 148 | 3,259 |
|  |  |  | 40 | 78.2 | 75.3 | 47.8 | 92.3 | 390 | 1,524 |
|  |  |  | 50 | 76.0 | 78.1 | 50.1 | 91.8 | 429 | 1,352 |
|  |  |  | 100 | 66.1 | 85.7 | 57.2 | 89.7 | 607 | 884 |
|  |  |  | 200 | 54.8 | 90.7 | 63.1 | 87.4 | 808 | 573 |
|  |  | Bacteria count | 20 | 96.9 | 57.3 | 39.6 | 98.5 | 55 | 2,639 |
|  |  |  | 50 | 94.4 | 73.0 | 50.3 | 97.8 | 101 | 1,666 |
|  |  |  | 100 | 91.3 | 80.9 | 58.1 | 97.0 | 156 | 1,178 |
|  |  |  | 200 | 86.7 | 87.6 | 67.0 | 95.8 | 237 | 764 |
|  |  |  | 250 | 84.8 | 89.2 | 69.4 | 95.3 | 271 | 668 |
| Surgery | Test strip | Nitrate | pos. | 40.2 | 97.9 | 83.4 | 86.2 | 944 | 126 |
|  |  | Leuk. esterase | pos. | 83.1 | 66.8 | 39.7 | 93.8 | 267 | 1,990 |
|  |  | Nitrate or Leukocyte esterase | pos. | 85.0 | 66.6 | 40.1 | 94.4 | 237 | 2,003 |
|  | Flow cytometry | Leukocyte count | 10 | 92.8 | 46.4 | 31.3 | 96.1 | 114 | 3,217 |
|  |  |  | 40 | 80.6 | 71.0 | 42.3 | 93.3 | 306 | 1,740 |
|  |  |  | 50 | 78.5 | 73.9 | 44.2 | 92.9 | 339 | 1,567 |
|  |  |  | 100 | 69.3 | 82.2 | 50.5 | 91.0 | 485 | 1,071 |
|  |  |  | 200 | 58.4 | 88.3 | 56.8 | 89.0 | 657 | 702 |
|  |  | Bacteria count | 20 | 97.8 | 62.0 | 40.3 | 99.1 | 35 | 2,283 |
|  |  |  | 50 | 95.3 | 76.6 | 51.7 | 98.4 | 74 | 1,405 |
|  |  |  | 100 | 91.6 | 84.4 | 60.7 | 97.5 | 132 | 935 |
|  |  |  | 200 | 87.3 | 89.9 | 69.4 | 96.4 | 201 | 607 |
|  |  |  | 250 | 85.5 | 91.3 | 72.2 | 96.0 | 229 | 520 |
| Intensive Care | Test strip | Nitrate | pos. | 27.8 | 98.8 | 78.5 | 89.3 | 304 | 32 |
|  |  | Leuk. esterase | pos. | 72.7 | 73.5 | 31.1 | 94.2 | 115 | 679 |
|  |  | Nitrate or Leukocyte esterase | pos. | 77.0 | 73.2 | 32.0 | 95.1 | 97 | 687 |
|  | Flow cytometry | Leukocyte count | 10 | 89.5 | 39.4 | 19.5 | 95.8 | 44 | 1,554 |
|  |  |  | 40 | 71.7 | 69.1 | 27.6 | 93.7 | 119 | 791 |
|  |  |  | 50 | 70.3 | 72.9 | 29.9 | 93.7 | 125 | 694 |
|  |  |  | 100 | 54.4 | 83.5 | 35.1 | 91.8 | 192 | 423 |
|  |  |  | 200 | 43.5 | 89.1 | 39.6 | 90.6 | 238 | 279 |
|  |  | Bacteria count | 20 | 97.4 | 55.5 | 26.5 | 99.2 | 11 | 1,140 |
|  |  |  | 50 | 95.5 | 73.8 | 37.4 | 99.0 | 19 | 672 |
|  |  |  | 100 | 90.7 | 83.0 | 46.8 | 98.2 | 39 | 435 |
|  |  |  | 200 | 87.2 | 89.2 | 57.0 | 97.7 | 54 | 277 |
|  |  |  | 250 | 85.0 | 90.4 | 59.4 | 97.4 | 63 | 245 |
| Gynecology/Obstetrics | Test strip | Nitrate | pos. | 21.6 | 98.8 | 85.5 | 79.0 | 621 | 29 |
|  |  | Leuk. esterase | pos. | 68.4 | 61.7 | 37.5 | 85.4 | 250 | 904 |
|  |  | Nitrate or Leukocyte esterase | pos. | 71.5 | 61.4 | 38.3 | 86.5 | 226 | 911 |
|  | Flow cytometry | Leukocyte count | 10 | 86.9 | 41.2 | 33.1 | 90.4 | 104 | 1,388 |
|  |  |  | 40 | 65.4 | 71.3 | 43.3 | 86.0 | 274 | 677 |
|  |  |  | 50 | 60.2 | 74.7 | 44.4 | 84.9 | 315 | 597 |
|  |  |  | 100 | 46.5 | 82.9 | 47.6 | 82.2 | 424 | 405 |
|  |  |  | 200 | 36.2 | 89.9 | 54.7 | 80.8 | 505 | 238 |
|  |  | Bacteria count | 20 | 98.4 | 29.3 | 31.8 | 98.2 | 13 | 1,669 |
|  |  |  | 50 | 96.5 | 44.2 | 36.7 | 97.4 | 28 | 1,317 |
|  |  |  | 100 | 93.4 | 55.4 | 41.3 | 96.2 | 52 | 1,053 |
|  |  |  | 200 | 88.5 | 66.0 | 46.6 | 94.5 | 91 | 802 |
|  |  |  | 250 | 86.6 | 69.4 | 48.7 | 93.9 | 106 | 722 |

| Children's Hosp. Basel | Test strip | Nitrate | pos. | 37.8 | 98.4 | 88.8 | 82.7 | 510 | 39 |
| --- | --- | --- | --- | --- | --- | --- | --- | --- | --- |
|  |  | Leuk. esterase | pos. | 84.3 | 64.3 | 43.8 | 92.5 | 129 | 888 |
|  |  | Nitrate or Leukocyte esterase | pos. | 87.0 | 64.1 | 44.4 | 93.7 | 107 | 892 |
|  | Flow cytometry | Leukocyte count | 10 | 92.1 | 43.5 | 35.0 | 94.3 | 65 | 1,405 |
|  |  |  | 40 | 79.3 | 69.9 | 46.5 | 91.1 | 170 | 747 |
|  |  |  | 50 | 77.0 | 72.8 | 48.3 | 90.5 | 189 | 675 |
|  |  |  | 100 | 68.2 | 81.6 | 55.0 | 88.6 | 261 | 458 |
|  |  |  | 200 | 60.1 | 87.1 | 60.6 | 86.9 | 327 | 320 |
|  |  | Bacteria count | 20 | 98.2 | 48.5 | 38.6 | 98.8 | 15 | 1,279 |
|  |  |  | 50 | 96.1 | 66.2 | 48.4 | 98.1 | 32 | 839 |
|  |  |  | 100 | 93.5 | 77.4 | 57.8 | 97.3 | 53 | 561 |
|  |  |  | 200 | 88.8 | 84.6 | 65.6 | 95.8 | 92 | 382 |
|  |  |  | 250 | 87.0 | 87.4 | 69.4 | 95.3 | 107 | 314 |
| Physicians, resident homes and others | Test strip | Nitrate | pos. | 39.4 | 97.1 | 87.5 | 75.8 | 215 | 20 |
|  |  | Leuk. esterase | pos. | 85.9 | 58.6 | 51.5 | 89.0 | 50 | 287 |
|  |  | Nitrate or Leukocyte esterase | pos. | 88.5 | 58.4 | 52.2 | 90.8 | 41 | 288 |
|  | Flow cytometry | Leukocyte count | 10 | 92.4 | 37.4 | 43.0 | 90.6 | 27 | 434 |
|  |  |  | 40 | 81.1 | 65.1 | 54.3 | 87.1 | 67 | 242 |
|  |  |  | 50 | 79.2 | 69.1 | 56.8 | 86.6 | 74 | 214 |
|  |  |  | 100 | 66.2 | 80.1 | 63.0 | 82.2 | 120 | 138 |
|  |  |  | 200 | 54.9 | 87.0 | 68.4 | 79.0 | 160 | 90 |
|  |  | Bacteria count | 20 | 98.0 | 45.7 | 48.1 | 97.8 | 7 | 376 |
|  |  |  | 50 | 95.8 | 65.8 | 58.9 | 96.8 | 15 | 237 |
|  |  |  | 100 | 93.2 | 75.9 | 66.5 | 95.6 | 24 | 167 |
|  |  |  | 200 | 90.4 | 83.7 | 74.0 | 94.5 | 34 | 113 |
|  |  |  | 250 | 89.6 | 85.0 | 75.4 | 94.1 | 37 | 104 |

Supplementary Table S 5 Individual calculation of sensitivity (SENS) and numbers of false negatives (FN) for samples of relevant monomicrobial growth (≥ 10^5^ CFU/ml of identified species) when test strips or flow cytometry was used to identify relevant bacteriuria using different cut-off values. Since species identification is not reliably available for samples of bacteriuria < 10^5^ CFU/ml (e.g. when classified as Enterobacteriaceae), specificity and other predictive values than SENS and FN are not available. Pos: positive test strip

| **Species** | **Method** | **Test Cut-off** | | **SENS (%)** | **FN** |
| --- | --- | --- | --- | --- | --- |
| *E.coli* (n=3489) | Test strip | Nitrate | pos. | 52.9 | 1645 |
|  |  | Leuk. esterase | pos. | 88.7 | 395 |
|  |  | Nitrate or Leukocyte esterase | pos. | 93.6 | 223 |
|  | Flow cytometry | Leukocyte count | 10 | 95.6 | 155 |
|  |  |  | 40 | 85.8 | 496 |
|  |  |  | 50 | 84.0 | 557 |
|  |  |  | 100 | 74.2 | 899 |
|  |  |  | 200 | 65.1 | 1217 |
|  |  | Bacteria count | 20 | 99.6 | 15 |
|  |  |  | 50 | 99.3 | 26 |
|  |  |  | 100 | 98.8 | 41 |
|  |  |  | 200 | 97.7 | 80 |
|  |  |  | 250 | 97.2 | 99 |
| *Klebsiella spp.* (n=763) | Test strip | Nitrate | pos. | 41.9 | 443 |
|  |  | Leuk. esterase | pos. | 89.8 | 78 |
|  |  | Nitrate or Leukocyte esterase | pos. | 92.8 | 55 |
|  | Flow cytometry | Leukocyte count | 10 | 97.1 | 22 |
|  |  |  | 40 | 87.3 | 97 |
|  |  |  | 50 | 85.3 | 112 |
|  |  |  | 100 | 76.5 | 179 |
|  |  |  | 200 | 64.0 | 275 |
|  |  | Bacteria count | 20 | 99.2 | 6 |
|  |  |  | 50 | 99.0 | 8 |
|  |  |  | 100 | 98.3 | 13 |
|  |  |  | 200 | 96.7 | 25 |
|  |  |  | 250 | 96.2 | 29 |
| *Lactobacillus spp.* (n=715) | Test strip | Nitrate | pos. | 1.3 | 706 |
|  |  | Leuk. esterase | pos. | 49.0 | 365 |
|  |  | Nitrate or Leukocyte esterase | pos. | 49.2 | 363 |
|  | Flow cytometry | Leukocyte count | 10 | 73.8 | 187 |
|  |  |  | 40 | 45.5 | 390 |
|  |  |  | 50 | 40.7 | 424 |
|  |  |  | 100 | 26.4 | 526 |
|  |  |  | 200 | 17.8 | 588 |
|  |  | Bacteria count | 20 | 98.7 | 9 |
|  |  |  | 50 | 96.1 | 28 |
|  |  |  | 100 | 89.8 | 73 |
|  |  |  | 200 | 79.6 | 146 |
|  |  |  | 250 | 75.8 | 173 |
| *Enterococcus faecalis* (n=483) | Test strip | Nitrate | pos. | 2.7 | 470 |
|  |  | Leuk. esterase | pos. | 69.2 | 149 |
|  |  | Nitrate or Leukocyte esterase | pos. | 69.4 | 148 |
|  | Flow cytometry | Leukocyte count | 10 | 86.1 | 67 |
|  |  |  | 40 | 66.5 | 162 |
|  |  |  | 50 | 64.2 | 173 |
|  |  |  | 100 | 50.7 | 238 |
|  |  |  | 200 | 41.0 | 285 |
|  |  | Bacteria count | 20 | 96.3 | 18 |
|  |  |  | 50 | 92.5 | 36 |
|  |  |  | 100 | 87.8 | 59 |
|  |  |  | 200 | 82.8 | 83 |
|  |  |  | 250 | 79.7 | 98 |
| *Gardnerella spp.* (n=316) | Test strip | Nitrate | pos. | 1.6 | 311 |
|  |  | Leuk. esterase | pos. | 49.7 | 159 |
|  |  | Nitrate or Leukocyte esterase | pos. | 50.0 | 158 |
|  | Flow cytometry | Leukocyte count | 10 | 74.4 | 81 |
|  |  |  | 40 | 40.8 | 187 |
|  |  |  | 50 | 37.0 | 199 |
|  |  |  | 100 | 23.7 | 241 |
|  |  |  | 200 | 15.2 | 268 |
|  |  | Bacteria count | 20 | 95.9 | 13 |
|  |  |  | 50 | 88.6 | 36 |
|  |  |  | 100 | 80.1 | 63 |
|  |  |  | 200 | 65.5 | 109 |
|  |  |  | 250 | 60.8 | 124 |
| *Pseudomonas aeruginosa* (n=223) | Test strip | Nitrate | pos. | 46.2 | 120 |
|  |  | Leuk. esterase | pos. | 88.8 | 25 |
|  |  | Nitrate or Leukocyte esterase | pos. | 91.0 | 20 |
|  | Flow cytometry | Leukocyte count | 10 | 96.9 | 7 |
|  |  |  | 40 | 88.8 | 25 |
|  |  |  | 50 | 85.2 | 33 |
|  |  |  | 100 | 75.3 | 55 |
|  |  |  | 200 | 67.3 | 73 |
|  |  | Bacteria count | 20 | 97.3 | 6 |
|  |  |  | 50 | 94.6 | 12 |
|  |  |  | 100 | 90.6 | 21 |
|  |  |  | 200 | 85.2 | 33 |
|  |  |  | 250 | 82.5 | 39 |
| *Enterococcus faecium* (n=213) | Test strip | Nitrate | pos. | 0.8 | 129 |
|  |  | Leuk. esterase | pos. | 64.6 | 46 |
|  |  | Nitrate or Leukocyte esterase | pos. | 64.6 | 46 |
|  | Flow cytometry | Leukocyte count | 10 | 86.9 | 17 |
|  |  |  | 40 | 60.8 | 51 |
|  |  |  | 50 | 57.7 | 55 |
|  |  |  | 100 | 43.1 | 74 |
|  |  |  | 200 | 30.8 | 90 |
|  |  | Bacteria count | 20 | 94.6 | 7 |
|  |  |  | 50 | 87.7 | 16 |
|  |  |  | 100 | 76.9 | 30 |
|  |  |  | 200 | 70.8 | 38 |
|  |  |  | 250 | 65.4 | 45 |


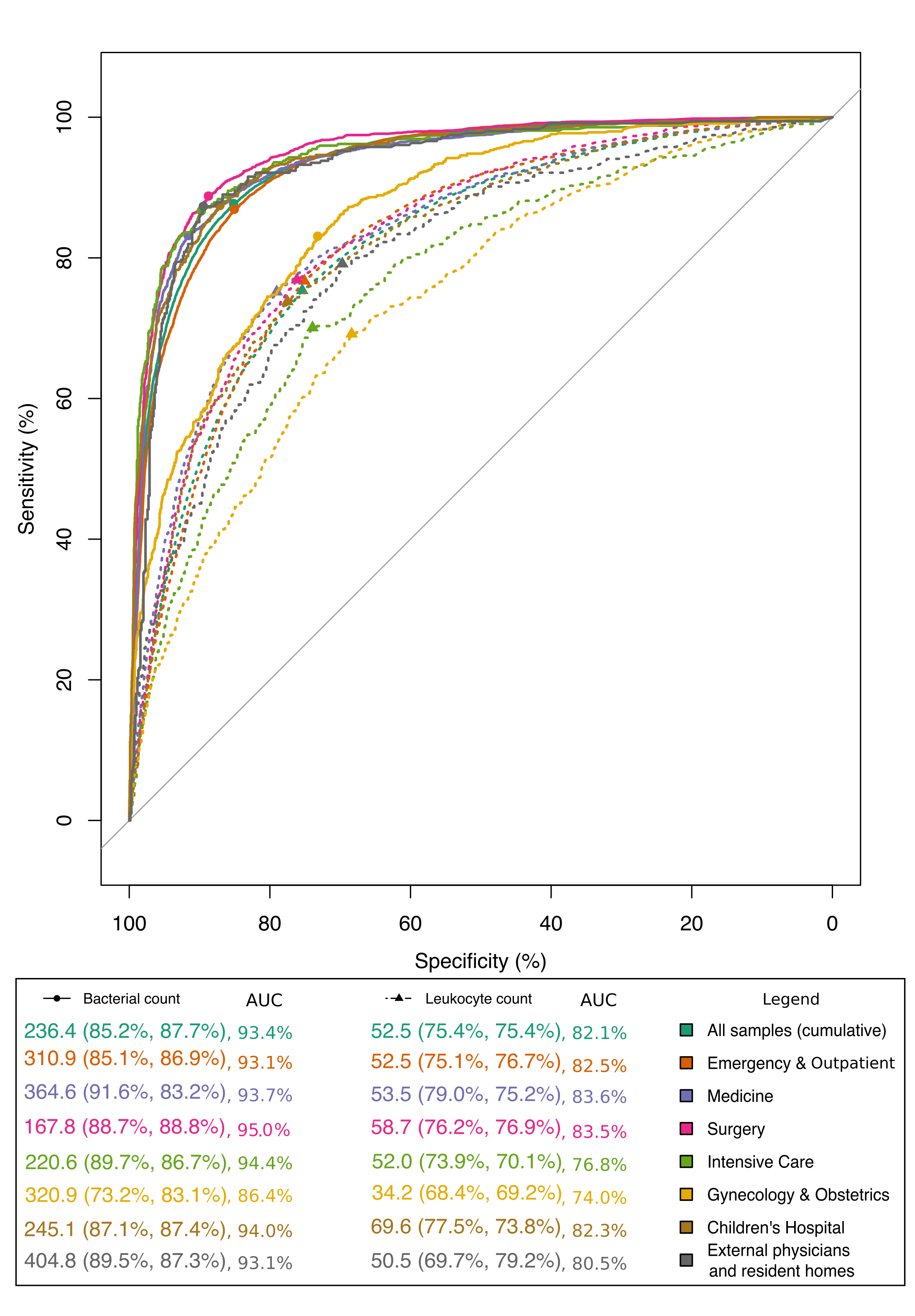


Supplementary Figure S 3 Receiver operating characteristic (ROC) curves of bacteria (continuous lines) and leukocyte counts (dotted lines) with the respective optimal calculated, unweighted cut-offs (and the respective specificity/sensitivity in brackets) and area under the ROC curves for all samples and for sub-analysis by the respective divisions ordering the test.

Supplementary Table S 6 Predictive values of epithelial cell counts measured by urine flow cytometry (UFC, a sum of squamous and small round cell counts) when used to identify contaminated samples. Sensitivity (SENS), specificity (SPEC), positive and negative predictive values (PPV and NPV), false negative (FN) and false positive results (FP), CFU: colony-forming units.

| Bacterial growth result | UFC cut-off "epithelial cells" | SENS (%) | SPEC (%) | PPV (%) | NPV (%) | FN | FP |
| --- | --- | --- | --- | --- | --- | --- | --- |
| Contaminated samples (mixed bacterial growth without a dominant bacterium),  n = 14701 | 3 | 69.00 | 40.30 | 34.08 | 74.41 | 4557 | 19623 |
|  | 4 | 61.24 | 48.72 | 34.82 | 73.76 | 5698 | 16855 |
|  | 10 | 35.51 | 72.78 | 36.85 | 71.62 | 9480 | 8946 |
| Contaminated samples + samples of bacterial growth <10^5^ CFU/ml,  n = 18826 | 3 | 69.15 | 41.73 | 43.73 | 67.38 | 5,808 | 16,749 |
|  | 4 | 61.18 | 50.11 | 44.54 | 66.34 | 7,308 | 14,340 |
|  | 10 | 34.98 | 73.63 | 46.49 | 63.36 | 12,240 | 7,581 |


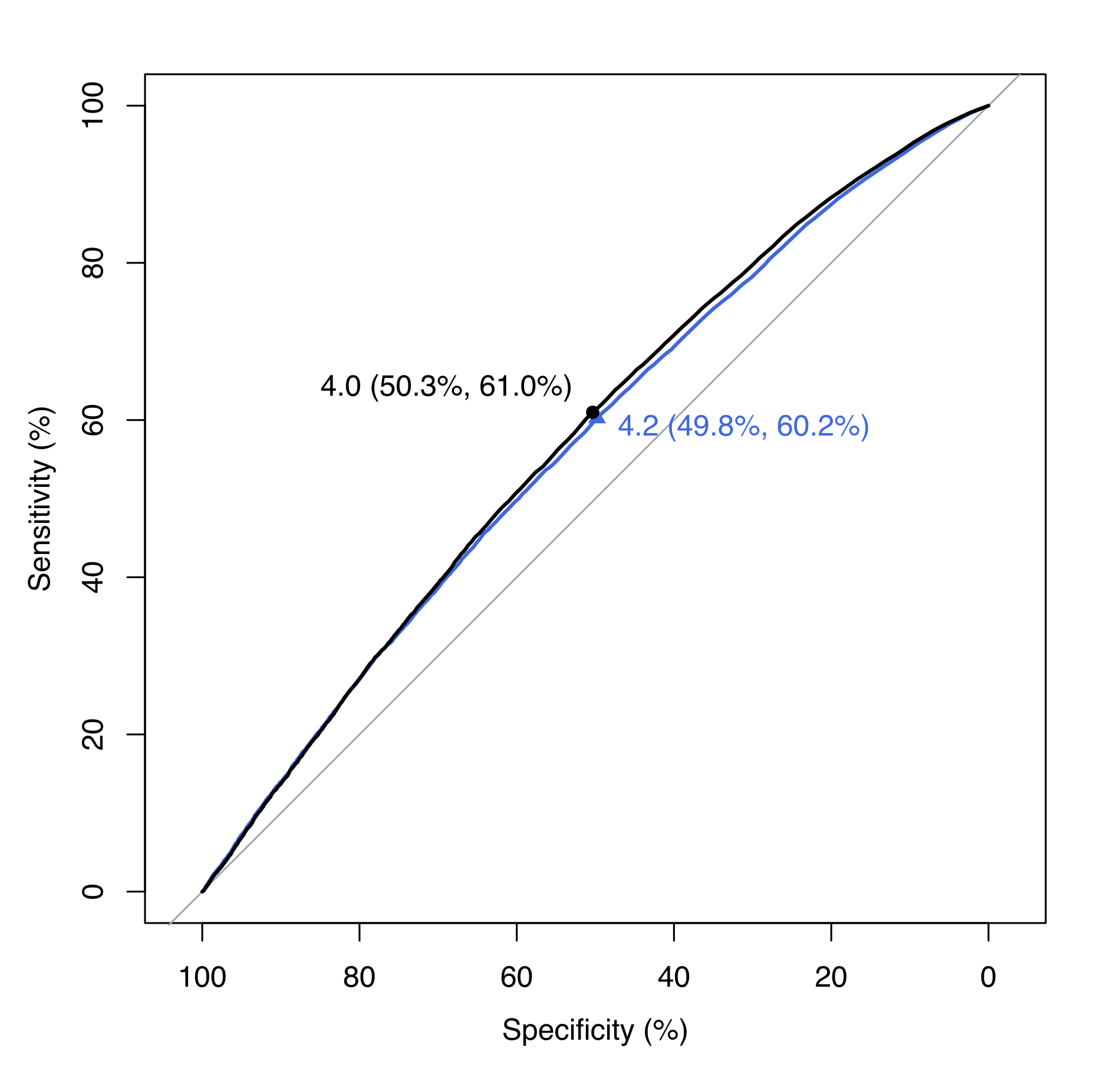


Supplementary Figure S 4 Receiver operating characteristic (ROC) curves of epithelial cell counts measured by flow cytometry with the respective optimal calculated, unweighted cut-offs (and the respective specificity/sensitivity in brackets) when used to discriminate all contaminated samples (polymicrobial growth without a dominant species, blue solid line, triangle) and all contaminated samples and additionally all samples with growth under 10^5^ colony forming units (black solid line, circle).


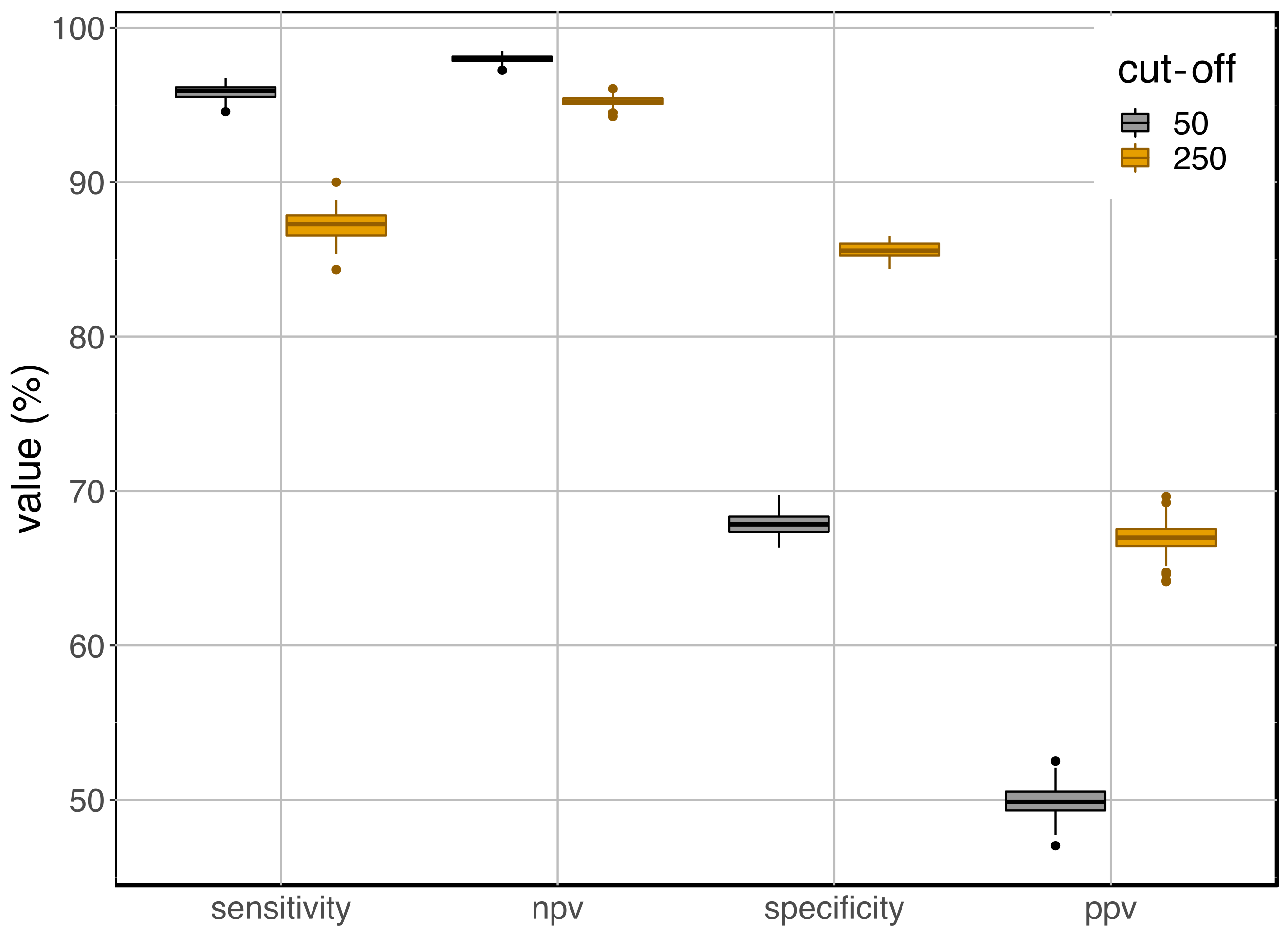


Supplementary Figure S 5 Performance of accuracy and predictive variables of the cut-offs selected in the relevant bacteriuria algorithm when assessed in the 10 times repeated 10-fold cross validation approach.

Supplementary Table S 7 Sensitivity (SENS), specificity (SPEC), positive and negative predictive values (PPV and NPV) of the cut-offs selected in the relevant bacteriuria algorithm when assessed in the 10 times repeated 10-fold cross validation approach.

| Cut-off | Variable | Median (1st - 3rd quartile) |
| --- | --- | --- |
| 50 | SENS | 95.9 (95.5 - 96.2) |
|  | NPV | 98.0 (97.9 - 98.1) |
|  | SPEC | 67.8 (67.4 - 68.3) |
|  | PPV | 49.9 (49.3 - 50.5) |
| 250 | SENS | 87.3 (84.3 - 87.9) |
|  | NPV | 95.2 (95.1 - 95.4) |
|  | SPEC | 85.6 (85.3 - 86.0) |
|  | PPV | 67.0 (66.4 - 67.5) |


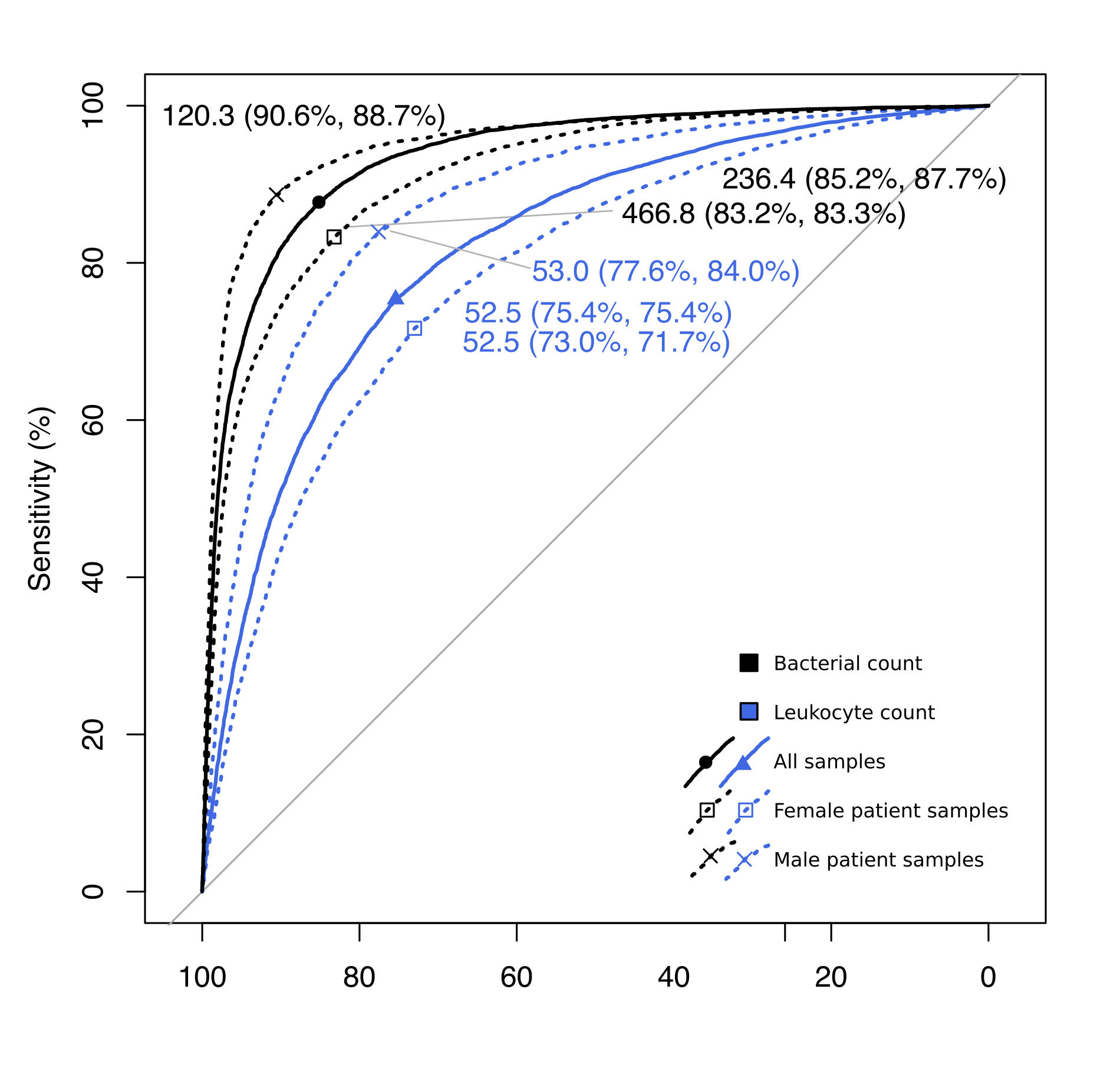


Supplementary Figure S 6 Receiver operating characteristic (ROC) curves of bacteria and leukocyte counts with the respective optimal calculated, unweighted cut-offs (and the respective specificity/sensitivity in brackets) for all samples, samples of male patients and samples of female patients.
